# Cardiorespiratory fitness in kidney transplant recipients: A pilot randomised controlled trial of structured home-based rehabilitation and a nested case-control analysis

**DOI:** 10.1177/02692155251408792

**Published:** 2025-12-30

**Authors:** Roseanne E Billany, Noemi Vadaszy, Stephanie Burns, Rafhi Chowdhury, Ella C Ford, Zahra Mubaarak, Gurneet K Sohansoha, Jian L Yeo, Abhishek Dattani, Alice C Cowley, Gaurav S Gulsin, Nicolette C Bishop, Alice C Smith, Gerry P McCann, Matthew PM Graham-Brown

**Affiliations:** 1 Division of Cardiovascular Sciences, 4488 University of Leicester, Leicester, UK; 2 Leicester Partnership for Kidney Health Research, Division of Cardiovascular Sciences, School of Medical Sciences, University of Leicester and NIHR Leicester Biomedical Research Centre, Leicester, UK; 3 Department of Renal Medicine, 4490 University Hospitals of Leicester NHS Trust, Leicester, UK; 4 Division of Public Health and Epidemiology, 4467 University of Leicester, Leicester, UK; 5 School of Humanities and Social Sciences, Leeds Beckett University, Leeds, UK; 6 School of Sport, Exercise and Health Sciences, 5156 Loughborough University, Loughborough, UK

**Keywords:** Kidney transplantation, home-based exercise, cardiovascular disease, cardiorespiratory fitness, cardiopulmonary exercise test

## Abstract

**Objectives:**

(1) Explore the effects of a 12-week home-based rehabilitation programme on cardiorespiratory fitness in kidney transplant recipients; (2) Compare cardiorespiratory fitness parameters in kidney transplant recipients and age-sex matched healthy volunteers to aid the justification for routine rehabilitation programmes.

**Design:**

Pilot randomised controlled trial with nested case-control.

**Setting:**

Home-based rehabilitation; hospital-based outcome assessments.

**Participants:**

Pilot randomised controlled trial: 50 stable kidney transplant recipients (>1 year post-transplant) (randomised 1:1; *n* = 25 control and *n* = 25 intervention). Nested case-control: 30 kidney transplant recipients and 30 healthy volunteers.

**Intervention:**

A 12-week home-based aerobic and resistance rehabilitation programme or guideline-directed care control.

**Main measures:**

Cardiorespiratory fitness measured by cardiopulmonary exercise testing.

**Results:**

Pilot randomised controlled trial: After adjusting for baseline, follow-up values were significantly greater in intervention compared to control for peak oxygen uptake (V̇O_2peak_) mL/kg/min, (+1.50, *p* = .03) and maximum workload (+8 W, *p* = .04) but not V̇O_2peak_ L/min or variables at the gas exchange threshold. Higher frequency of aerobic exercise sessions was associated with greater improvements in cardiorespiratory fitness (*R*_2_ = .252, *p* = .040).

Nested case-control: V̇O_2peak_ was reduced in kidney transplant recipients compared to healthy volunteers (18.81 ± 4.61 vs 24.06 ± 5.72 mL/kg/min; *p* < .01), as was V̇O_2_ at the gas exchange threshold (11.70 ± 2.67 vs 14.47 ± 3.39 mL/kg/min; *p* < .01).

**Conclusions:**

A 12-week home-based rehabilitation programme induced a significant improvement in some cardiorespiratory fitness variables and higher frequency of aerobic exercise associated with greater improvements. Cardiorespiratory fitness is significantly impaired in kidney transplant recipients compared to age-sex-matched healthy volunteers. Together, these findings highlight the clinical importance of promoting aerobic exercise and the integration of rehabilitation programmes into routine care for this population.

**Trial registration:**

ClinicalTrials.gov, NCT04123951 (https://clinicaltrials.gov/study/NCT04123951).

## Introduction

Physical inactivity remains a key, modifiable driver of cardiovascular disease in kidney transplant recipients, with only 27% of the population considered physically active for health.^1^ Whilst kidney transplantation confers a significant survival advantage compared to remaining on dialysis,^2^ cardiovascular disease remains a leading cause of morbidity and mortality and is a factor linked to all-cause graft loss.^3^ The reasons for this are complex, but relate to the clustering of traditional and non-traditional risk factors for cardiovascular disease. Some of these are present pre-transplantation, including hypertension, dyslipidaemia, obesity, diabetes, physical inactivity, dialysis and smoking, whilst others emerge following transplantation driven by the requirement to take immunosuppressive therapies, including accelerated weight gain, metabolic syndrome, and new onset diabetes after transplant.^4^

Cardiorespiratory fitness is a strong, independent predictor of all-cause and cardiovascular mortality^5,6^ and is objectively quantified using gold-standard cardiopulmonary exercise testing. Whilst oxygen uptake (V̇O_2peak_), a key variable of cardiorespiratory fitness, does improve 12 months after kidney transplantation compared to pre-transplantation, it remains impaired when compared to healthy participants.^7,8^ Confirmation is required of these results due to the considerable differences in age between groups in these studies, making conclusive comparisons challenging. Age and sex influence V̇O_2peak_ profoundly, particularly age, where a slow decline occurs across the lifespan.

Exercise interventions (although of limited quality) have been shown to lead to significant improvements in cardiorespiratory fitness in kidney transplant recipients.^9^ Each 1 metabolic equivalent (1-MET; ∼3.5 mL/kg/min) increase in cardiorespiratory fitness has been associated with a 19% lower cardiovascular disease mortality risk amongst patients with cardiovascular disease.^10^ Despite this, rehabilitation for kidney transplant recipients is not commonplace. In-centre, supervised programmes are not realistically deliverable in the current climate, and few studies have explored the effectiveness of home-based exercise for improving cardiorespiratory fitness in kidney transplant recipients.

Given that physical activity and exercise levels remain low following kidney transplantation for many patients, there is the potential that an effective home-based programme of exercise and physical activity post-transplantation will improve cardiorespiratory fitness and reduce cardiovascular disease risk.

In this study, we aimed to:

1. Explore the effects of a home-based rehabilitation programme on cardiorespiratory fitness in kidney transplant recipients
2. Compare cardiorespiratory fitness parameters in kidney transplant recipients and age-sex-matched healthy volunteers to aid the justification for routine rehabilitation programmes

## Methods

Data from two clinical trials were used in this study. Data for kidney transplant recipients were taken from the ECSERT study (NCT04123951). Data for healthy volunteers were collected from the PREDICT study (NCT03132129). Both studies were registered prospectively and ethical approval was granted by East Midlands – Nottingham and West Midlands – Solihull research ethics committees respectively (ref 19/EM/0209; ref 17/WM/0192). The University of Leicester was the sponsor for both trials and participants provided written informed consent. This manuscript has been written in alignment with the Consolidated Standards Of Reporting Trials (CONSORT) extension for pilot and feasibility trials.^11^

### Randomised controlled trial of a home-based rehabilitation intervention in kidney transplant recipients (ECSERT)

The ECSERT trial was a pilot prospective, randomised, open-label, blinded endpoint study performed at one kidney unit in the United Kingdom between March 2020 and February 2023. Stable adult kidney transplant recipients >1 year post-transplantation were eligible to participate. Full details of the inclusion and exclusion criteria and the trial protocol are as previously published.^12^ The primary aim was to assess the feasibility of a 12-week home-based exercise programme in kidney transplant recipients and as such no power calculation was completed *a-priori* for the secondary analyses presented here. Full feasibility outcomes have been previously published,^13^ and data presented here are not previously published.

Following baseline assessment, kidney transplant recipients were randomly allocated (1:1) to either (1) a 12-week home-based combined aerobic and resistance exercise programme or (2) control (receiving guideline-directed care). Randomisation was blocked (using computer-generated random permuted blocks with allocation concealment performed by the Clinical Trials Facilitator; https://www.sealedenvelope.com/simple-randomiser/v1/) to ensure periodic balancing. Given the nature of the intervention, it was not possible for participants to be blinded to their allocation or assessments.

The 12-week, home-based, structured exercise programme included aerobic and resistance training (4–5 sessions in total per week). Participants were advised to complete a warm-up and cool-down prior to and following each session, respectively. Participants continued to receive usual clinical care. The aerobic component of the programme was walking, jogging, cycling, or similar, depending on resources available and participant preference. Participants were asked to complete 2–3 sessions per week using a rating of perceived of exertion of 13–15 (somewhat hard-hard) for 20–30 min. The resistance component of the exercise programme included a combination of 6–8 exercises per session chosen by the participant from a pool of twelve exercises targeting upper and lower body and core muscle groups, using free weights and/or resistance bands. The chosen pool of exercises included: squat, hip abduction, lunge, calf-raise, side-lunge, bicep-curl, bent-over row, reverse-fly, lateral-raise, chest-press, side-bends, and standing trunk rotation. Participants were asked to complete 6–8 resistance exercises twice a week (but not on consecutive days to allow appropriate recovery). Initially they were advised to complete 1–2 sets of 10 repetitions (at 60% 1-repetition maximum), gradually increasing to 3–6 sets of 10 repetitions with a minimum of 30 s rest between sets.

Participants were provided with an exercise diary which included additional instructions, dumbbells and resistance bands, and access to educational and instructional videos. Participants received a telephone call from a member of the research team every two weeks to discuss the progression of the exercise, address issues, and monitor adherence.

Participants in the control group were advised to be physically active and undertake exercise aligned to current guidelines.^14^ Participants were reminded to attend any scheduled clinic appointments and to take prescribed medication as advised. Advice about exercise and activity was reiterated to patients in the control group to ensure the intervention was being appropriately compared to best-practice guideline-directed care.

#### Outcome measure assessments

At baseline, participants performed a cardiopulmonary exercise test utilising a symptom-limited incremental ramp protocol (one-minute workload increments based on participant characteristics^15^) performed on a stationary electronically braked cycle ergometer with simultaneously expired gas analysis. Test data were considered usable if the respiratory exchange ratio was ≥1.00. A continuous 12-lead electrocardiogram was monitored throughout, with blood pressure recording at 2-min intervals. The test was conducted in the presence of a physician or cardiac nurse specialist. Peak oxygen uptake (V̇O_2peak_) was determined as the highest recorded value after data smoothing (30 s rolling mean). Gas exchange threshold was determined by two independent investigators (Roseanne E Billany and Noemi Vadaszy) using the V̇CO_2_-V̇O_2_ relationship (where V̇CO_2_ is volume of expired carbon dioxide), the ventilatory equivalents plotted against V̇O_2_ (L/min), and the end-tidal gas tensions plotted against V̇O_2_ (L/min).^16^ Predicted V̇O_2_ was determined in accordance with recommendation from the American Heart Association^17^ using the equations proposed by Wasserman and Hansen accounting for age, sex, and weight.^15,18^ Cardiorespiratory fitness categories were determined as described by the American Heart Association.^16^ Heart rate recovery was calculated as the difference between maximal exercise heart rate and heart rate 1 min into recovery. Other variables included within the analysis were defined as: V̇E (volume of expired air (or inspired air) per minute), V̇E/V̇CO_2_ slope (represents matching of the ventilation and perfusion within the pulmonary system), V̇E/V̇O_2_ (reflective of the ventilatory cost of O_2_ uptake), PETCO_2_ (CO_2_ partial pressure at the end of expiration also representative of the matching of the ventilation and perfusion), and O_2_ pulse (ratio between V̇O_2_ and heart rate and a non-invasive reflection of stroke volume). Follow-up assessments with repeat cardiopulmonary exercise tests were conducted for exercise and control groups within 7 days of completing the 12-week exercise or control period.

### Nested case-control

Kidney transplant recipients from the ECSERT study and healthy volunteers from the PREDICT study were matched for exact age and sex (1:1; ± 1 year). Healthy volunteers were recruited through the PREDICT study between November 2017 and 2021. PREDICT is a single-centre, case-control study comprising extensive cardiovascular phenotyping in adults with and without Type 2 diabetes. Inclusion and exclusion criteria have been previously reported.^19^ Only data from healthy volunteers in PREDICT are incorporated within this study and key criteria for inclusion were: adults aged 18–75 years with no prior history of cardiovascular disease and no diagnosis of diabetes mellitus or impaired glucose tolerance were recruited. Additional exclusion criteria within this cohort included: uncontrolled hypertension (blood pressure >160/100 mmHg) and conditions that may limit exercise capacity or be associated with subclinical cardiac dysfunction. Healthy volunteers performed an identical cardiopulmonary exercise test protocol as outlined above.

### Demographic and clinical data

For kidney transplant recipients, demographics, medical history, anthropometric measures, and blood samples were collected at the ECSERT study baseline visit. Blood samples were collected to assess diabetes control, lipids, liver and kidney function. For healthy volunteers, demographics, medical history, anthropometric measures, and blood samples (as above) were collected at the PREDICT study baseline visit.

### Data collection, management and analysis

Data from all time points were collected in case report forms by the trial team. All data was entered into a secure database only accessible on password-protected computers at the University Hospitals of Leicester and the University of Leicester by relevant members of the study team. No identifying information was kept in electronic form. All source data and original participant identities were kept in a locked office in the trial site file at the University Hospitals of Leicester.

Data were assessed for normality using histograms, Q-Q plots, and the Shapiro-Wilk test. Continuous data are expressed as mean (± standard deviation (SD)) if normally distributed or median (interquartile range) if not. Statistical analysis was performed using IBM SPSS Statistics for Windows, Version 26.0 (Armonk, NY, USA: IBM Corp. Released 2019). A *p* value <0.05 was considered statistically significant unless otherwise stated.

To investigate the differences between interventions in the randomised controlled trial, analysis of co-variance adjusted for baseline value was used. Assumptions of homogeneity of regression slopes, homogeneity of variances, and normality were checked. Partial Eta squared (η^2^) are reported for effect size and are interpreted as: small (0.01), medium (0.06), or large (0.14) effect. Bivariate linear regression models were used within the intervention group to determine the association between the total number of exercise sessions recorded during the 12-week programme and the change at 12 weeks (follow-up minus baseline) in cardiorespiratory fitness.

Healthy volunteer and kidney transplant recipients’ variables in the nested case-control study were compared by independent *t*-tests, or Mann-Whitney tests as appropriate. Categorical variables were presented as absolute values and relative frequency and were compared using the χ^2^ test or Fisher exact test as appropriate. Differences in PETCO_2_ between groups at rest, gas exchange threshold, and peak were compared using a 2 × 3 analysis of variance. Fishers least significant difference and Bonferroni corrections were used for post-hoc analysis of the main effect of group and time, respectively.

## Results

### The ECSERT randomised controlled trial

**Figure 1.**
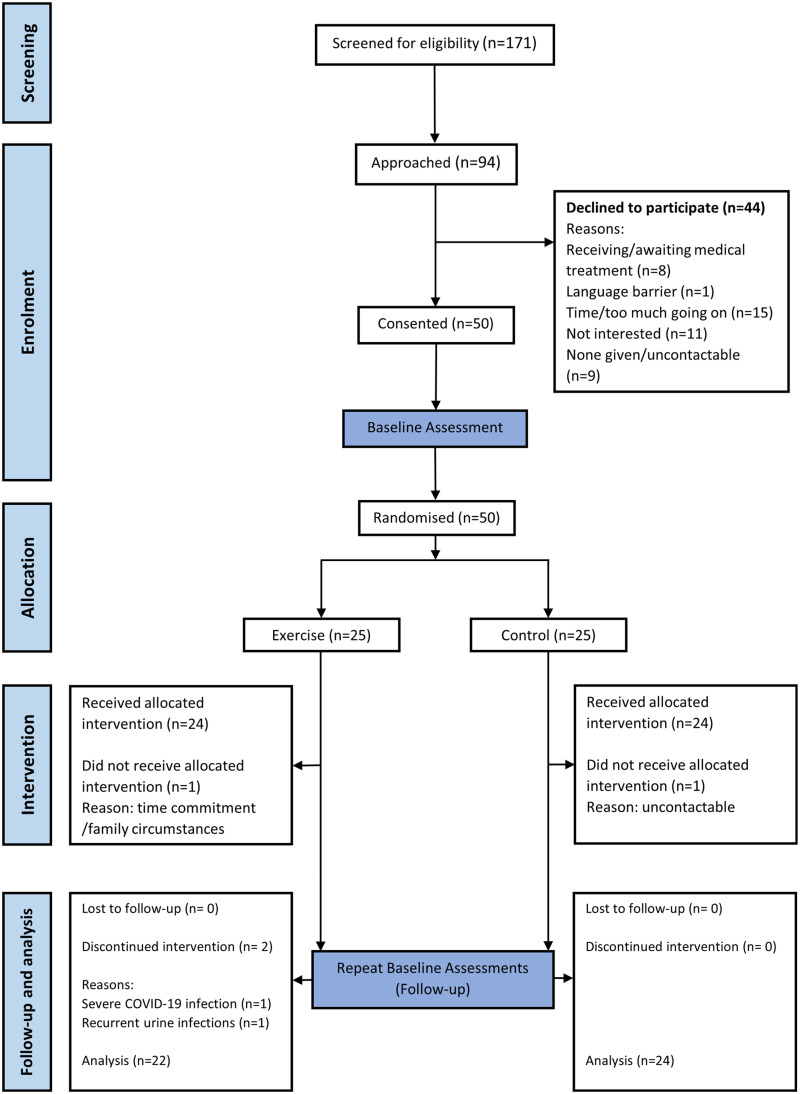
CONSORT diagram for the flow of participants through the ECSERT trial.

Fifty kidney transplant recipients were recruited and completed baseline cardiopulmonary exercise test assessments as part of the ECSERT study, with 25 patients randomised to intervention and 25 to control groups as per trial recruitment target. Baseline demographic characteristics of intervention and control groups are shown in Table 1. Participants were 50 ± 14 years old with an average estimated glomerular filtration rate (eGFR) of 61 ± 20 mL/min/1.73 m^2^. Sixteen participants (32%) were of a non-White British background and 27 (54%) were female. Forty-six participants (92%, 95% confidence interval (CI) [84.5, 99.5]) completed baseline and completed follow-up assessments (attrition 8%, 95% CI [0.5, 15.5]; Figure 1).

**Table 1.** ECSERT baseline demographics and clinical characteristics.

Variable	Intervention (*n* = 25)	Control (*n* = 25)	All (*n* = 50)
Age (years)	49 ± 13	51 ± 15	50 ± 14
Sex, *n* (%)			
Male	10 (40)	13 (52)	23 (46)
Female	15 (60)	12 (48)	27 (54)
Ethnicity, *n* (%)			
White/White British	16 (64)	18 (72)	34 (68)
South Asian	8 (32)	6 (24)	14 (28)
Caribbean	0 (0)	1 (4)	1 (2)
Other ethnic	1 (4)	0 (0)	1 (2)
KRT (months)^a^	46 (20–107)	46 (26–165)	46 (25–115)
eGFR (mL/min/1.73 m^2^)	60 ± 20	61 ± 21	61 ± 20
Body mass (kg)	84 ± 26	80 ± 14	82 ± 21
Smoking status, *n* (%)			
Current	3 (12)	1 (4)	4 (8)
Previous	5 (20)	11 (44)	16 (32)
Co-morbidities, *n* (%)			
Hypertension	24 (96)	23 (92)	47 (94)
Type II diabetes	5 (20)	4 (16)	9 (18)
Heart condition	0 (0)	1 (4)	1 (2)
Hyperlipidaemia	14 (56)	14 (56)	28 (56)
Medication, *n* (%)			
CNI	25 (96)	25 (100)	49 (98)
mTOR inhibitor	1 (4)	0 (0)	1 (2)
Mycophenolic acid	24 (96)	21 (84)	45 (90)
Steroid	19 (76)	19 (76)	38 (76)
Antihypertensive	22 (88)	22 (88)	44 (88)
Diabetes pharmacotherapy	5 (20)	4 (16)	9 (18)
Statin	12 (48)	11 (44)	23 (46)
Vitamin D/calcium	12 (48)	14 (56)	26 (52)

Notes: Unless otherwise indicated, values for categorical variables are expressed as integer (% of *n*); values for continuous variables as *M* ± SD.

KRT: kidney replacement therapy; eGFR: estimated glomerular filtration rate; CNI: calcineurin inhibitor; mTOR: mammalian target of rapamycin; IQR: interquartile range.

a Median (IQR).

**Figure 2.**
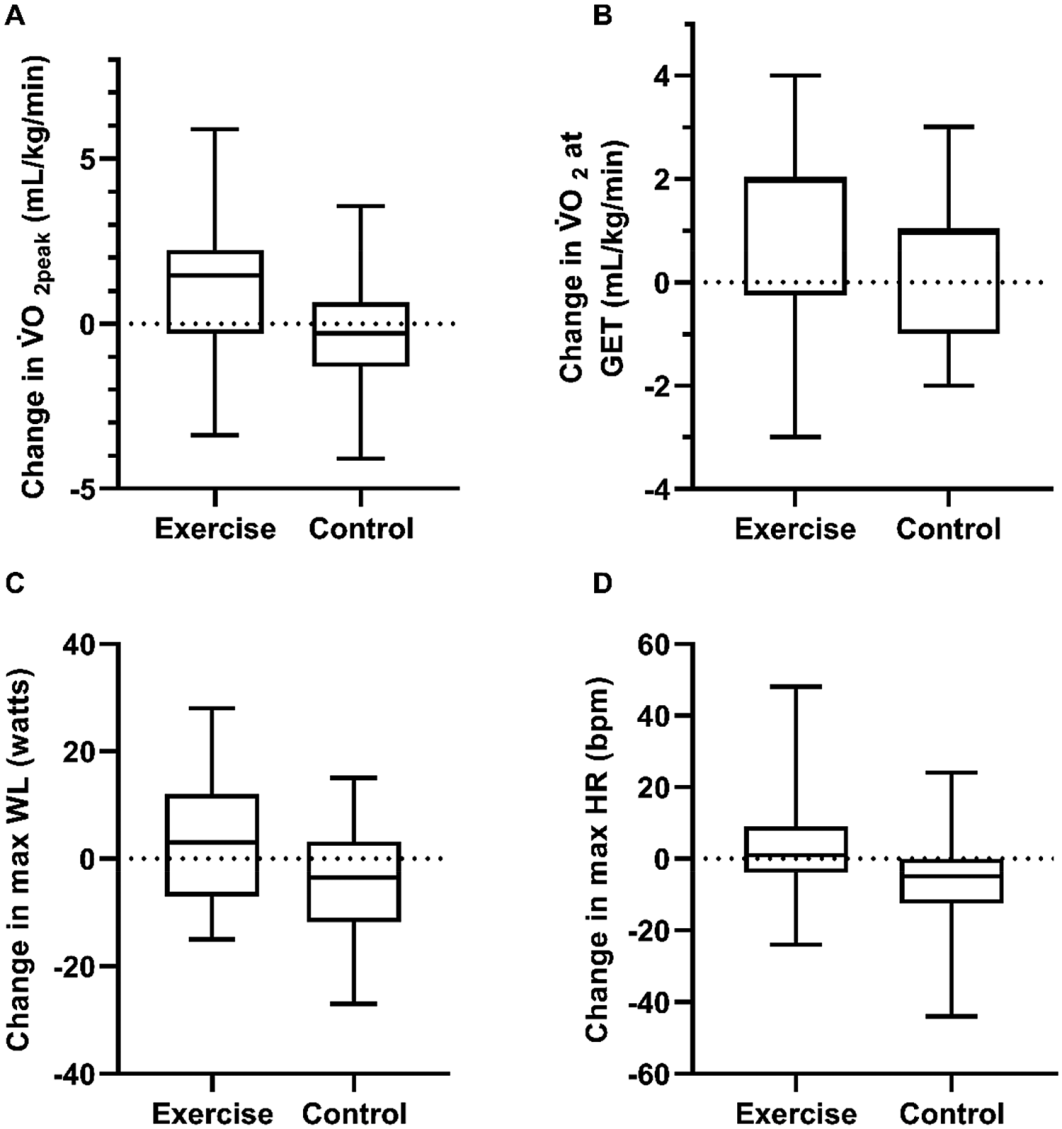
Change in variables over the study period for intervention and control in V̇O_2peak_ (a, mL/kg/min); V̇O_2_ at GET (b, mL/kg/min); max WL (c, watts); max HR (d, bpm).

GET: gas exchange threshold; HR: heart rate; V̇O_2_: oxygen uptake; WL: workload. Error bars show the maximum and minimum values.

Table 2 and Figure 2 show the changes in measures of cardiorespiratory fitness between intervention and control groups over the study period. After adjusting for baseline values, follow-up values at peak exercise were significantly greater in the intervention group compared to control for VO_2peak_ mL/kg/min, *F*(1, 37) = 5.20, *p* = .028, max heart rate (HR), *F*(1, 37) = 4.35, *p* = .044, and max workload, *F*(1, 37) = 4.83, *p* = .034, but not V̇O_2peak_ L/min *F*(1, 37) = 2.60, *p* = .115. Post-intervention V̇O_2peak_ mL/kg/min, after baseline adjustment, was 1.50 mL/kg/min (95% CI [0.16, 2.81]) greater in the intervention versus the control group. There were no other statistically significant differences in peak or recovery values. After adjusting for baseline values, there were no significant differences between groups for cardiorespiratory fitness variables at gas exchange threshold (Table S1 in the Supplemental Materials).

**Table 2.** Analysis of unadjusted and adjusted mean difference between intervention and control for cardiorespiratory fitness variables at peak exercise and recovery.

Variable	*n*	Baseline (*M* ± SD)	Follow-up		Adjusted mean difference B − A (CI of Diff) *p* value (η^2^)
			Unadjusted (*M* ± SD)	Adjusted (*M* ± SE)	
Peak					
V̇O_2peak_ (mL/kg/min)					
Intervention	19	20.2 ± 5.2	21.1 ± 5.9	21.8 ± 0.5	1.5 (0.2, 2.8) **0.03 (0.12)**
Control	21	21.4 ± 7.0	20.9 ± 7.7	20.3 ± 0.5	
V̇O_2peak_ (L/min)					
Intervention	19	1.7 ± 0.6	1.7 ± 0.6	1.7 ± 0.1	0.1 (−0.0, 0.2) 0.12 (0.06)
Control	21	1.7 ± 0.5	1.7 ± 0.6	1.6 ± 0.1	
V̇O_2peak_pred (%)					
Intervention	19	80.1 ± 21.3	84.1 ± 21.9	87.6 ± 2.3	4.0 (−2.8, 10.7) 0.24 (0.04)
Control	21	87.2 ± 23.9	86.7 ± 24.8	83.6 ± 2.3	
Max WL (watts)					
Intervention	20	124 ± 45	128 ± 46	129 ± 3	8 (1, 15) **0.03 (0.12)**
Control	20	126 ± 39	122 ± 43	121 ± 3	
Max HR (bpm)					
Intervention	19	148 ± 26	150 ± 26	149 ± 3	10 (0, 19) **0.04 (0.12)**
Control	21	144 ± 28	138 ± 30	139 ± 3	
V̇CO_2_ (L/min)					
Intervention	18	1.9 ± 0.6	1.9 ± 0.7	1.8 ± 0.1	−0.0 (−0.4, 0.2) 0.57 (0.01)
Control	19	1.6 ± 0.5	1.8 ± 0.7	1.9 ± 0.1	
V̇E (L/min)					
Intervention	19	68.6 ± 25.4	70.0 ± 32.4	72.2 ± 3.8	3.1 (−7.6, 13.6) 0.56 (0.00)
Control	21	73.0 ± 22.1	71.1 ± 23.3	69.1 ± 3.6	
V̇E/V̇CO2 slope					
Intervention	18	34.0 ± 7.4	35.7 ± 8.0	36.8 ± 1.2	2.0 (−1.3, 5.4) 0.23 (0.04)
Control	20	36.3 ± 9.4	35.7 ± 9.3	34.7 ± 1.1	
V̇E/V̇O2 ratio					
Intervention	18	38.3 ± 6.3	39.2 ± 7.5	38.9 ± 1.1	0.7 (−2.4, 3.8) 0.65 (0.01)
Control	19	37.6 ± 5.2	37.9 ± 5.5	38.2 ± 1.1	
O_2_ pulse (ml/beat)					
Intervention	15	10.8 ± 3.6	11.0 ± 3.5	11. ± 0.4	0.2 (−1.3, 1.5) 0.77 (0.00)
Control	12	12.3 ± 2.9	12.2 ± 3.5	11.4 ± 0.5	
RO_2_ pulse (mL/kg/beat)					
Intervention	15	0.13 ± 0.03	0.14 ± 0.03	0.14 ± 0.01	0.01 (−0.02, 0.02) 0.82 (0.00)
Control	12	0.15 ± 0.32	0.15 ± 0.04	0.14 ± 0.01	
Recovery					
HR recovery (%)					
Intervention	15	13.4 ± 4.5	18.7 ± 11.0	18.8 ± 2.8	2.6 (−4.2, 12.4) 0.50 (0.02)
Control	17	14.4 ± 5.3	16.4 ± 11.0	16.2 ± 2.8	
HR recovery (bpm)					
Intervention	15	20.3 ± 7.9	28.3 ± 17.9	28.7 ± 4.5	4.7 (−8.1, 17.5) 0.46 (0.02)
Control	17	21.9 ± 9.3	24.4 ± 17.6	24.0 ± 4.5	

A: control; B: intervention; V̇O_2_: oxygen uptake; pred: predicted; WL: workload; HR: heart rate; V̇CO_2_: volume of expired carbon dioxide; V̇E: volume of expired air per minute; R: relative. Values in bold indicate significance *p* < 0.05.

Total number of self-reported exercise sessions (aerobic and resistance) was not a significant predictor of change in V̇O_2peak_ mL/kg/min, *F*(1, 15) = 3.98, *R*_2_ = *.*210*, p* = .065, or V̇O_2peak_ L/min *F*(1, 15) = 3.74, *R*_2_ = .200, *p* *=* .072 (Figure S1, A and B, in the Supplemental Materials). Total self-reported number of aerobic training sessions was a significant predictor of V̇O_2peak_ L/min, *F*(1, 15) = 5.05, *R*_2_ *=* *.*252*, p* *=* .040, but not V̇O_2peak_ mL/kg/min *F*(1, 15) = 4.27, *R*_2_ = .222, *p* = .057 (Figure S1, C and D, in the Supplemental Materials). Self-reported number of resistance training sessions was not a significant predictor of change in any cardiorespiratory fitness outcomes.

### Nested case-control study

Thirty kidney transplant recipients were included from the ECSERT study. Thirty healthy volunteers were selected from the PREDICT study matched for age and sex. The baseline and clinical demographics of the kidney transplant recipients and healthy volunteers are presented in Table 3. Kidney function was significantly lower in kidney transplant recipients than healthy volunteers (mean eGFR difference = −25.80 ml/min/1.73m^2^, 95% CI [−33.12, −18.48). Kidney transplant recipients had significantly higher prevalence of hypertension and dyslipidaemia, and accordingly were prescribed more medications (statins, antihypertensives, steroids, and diabetes therapy). Diastolic blood pressure was lower in kidney transplant recipients than in healthy volunteers (mean difference = −7 mmHg, 95% CI [−12, −2]).

**Table 3.** Clinical and demographic characteristics for case-control.

Variable	Healthy volunteers (*n* = 30)	Kidney transplant recipients (*n* = 30)	*p* value
Demographics			
Age (years)	61 ± 7	61 ± 8	.812
Sex, *n* (%)			
Male	14 (47)	14 (47)	1.000
Female	16 (53)	16 (53)	
Ethnicity, *n* (%)			
White British	22 (73)	21 (70)	.554
Asian or Asian British	8 (27)	7 (23)	
Caribbean	0 (0)	1 (3)	
Other ethnic	0 (0)	1 (3)	
Anthropometrics			
Height (cm)	169 ± 9	168 ± 11	.515
Weight (kg)	77 ± 18	81 ± 20	.371
BMI (kg/m^2^)	26.6 ± 4.8	28.7 ± 5.7	.127
Haemodynamics			
Heart rate (bpm)	70 ± 12	73 ± 10	.394
SBP (mmHg)	139 ± 20	134 ± 22	.428
DBP (mmHg)	85 ± 8	78 ± 10	.**003**
Blood chemistry			
eGFR (mL/min/1.73 m^2^)	81.3 ± 9.2	55.5 ± 17.8	**<**.**001**
Medical history			
KRT (months)^a^	N/A	50 (27–140)	N/A
Previous dialysis, *n* (%)	N/A	16 (53)	N/A
Hypertension, *n* (%)	5 (17)	28 (93)	**<**.**001**
Dyslipidaemia, *n* (%)	3 (10)	24 (80)	**<**.**001**
Smoking status, *n* (%)			
Never	16 (53)	20 (67)	.430
Current	0 (0)	0 (0)	
Previous	14 (47)	10 (33)	
Medication, *n* (%)			
Statin	4 (13)	22 (73)	**<**.**001**
Diabetes pharmacotherapy	0 (0)	9 (30)	.**002**
Antihypertensive	4 (13)	29 (97)	**<**.**001**
Steroid	1 (3)	20 (67)	**<**.**001**
Mycophenolic acid	N/A	26 (87)	N/A
CNI	N/A	29 (97)	N/A
mTOR inhibitor	N/A	1 (3)	N/A

Notes: Unless otherwise indicated, values for categorical variables are expressed as integer (% of *n*); values for continuous variables as *M* ± SD.

BMI: body mass index; SBP: systolic blood pressure DBP: diastolic blood pressure; eGFR: estimated glomerular filtration rate; KRT: kidney replacement therapy; N/A: not applicable; CNI: calcineurin inhibitor; mTOR: mammalian target of rapamycin; IQR: interquartile range. Values in bold indicate significance *p*<0.05.

a Median (IQR).

**Figure 3.**
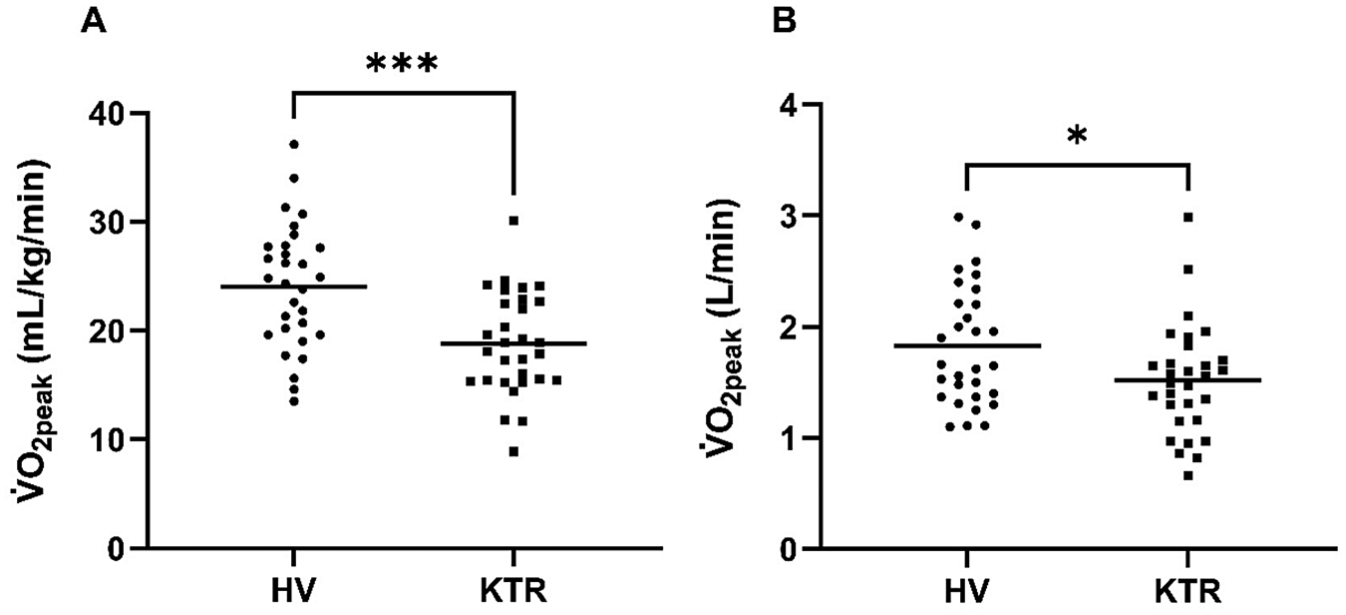
Distribution of values for cardiorespiratory fitness parameters in healthy volunteers and kidney transplant recipients. (a, V̇O_2peak_ [mL/kg/min]; b, V̇O_2peak_ [L/min]).

Observed frequencies and percentages of participants in each cardiorespiratory fitness category can be found in Table S2 in the Supplemental Materials. In summary, more kidney transplant recipients are within the low and fair categories compared to average and good in healthy volunteers. Cardiopulmonary outcomes are shown in Table 4. Cardiorespiratory fitness (V̇O_2peak_) was significantly reduced in kidney transplant recipients when compared to healthy volunteers in absolute terms and relative to body weight (Figure 3). Kidney transplant recipients on average achieved 86% of age-sex predicted maximum V̇O_2_ compared to 106% in healthy volunteers. Peak power output was significantly less in kidney transplant recipients than in healthy volunteers (113 ± 38 vs 147 ± 42 W; *p* = .006). Lower V̇O_2_ at the gas exchange threshold was observed in kidney transplant recipients compared to healthy volunteers (mean difference = −2.77 mL/kg/min, 95% CI [−4.35, −1.18]). As a percentage of predicted maximum, gas exchange threshold occurred at a value 10% lower in kidney transplant recipients than healthy volunteers.

V̇O_2_: oxygen uptake; HV: healthy volunteers; KTR: kidney transplant recipients.

*p ≤ .05, ***p ≤ .001.

**Table 4.** Cardiopulmonary exercise testing variables between kidney transplant recipients and healthy volunteers.

Variable	Healthy volunteers (*n* = 30)	Kidney transplant recipients (*n* = 30)	*p* value
Rest			
V̇O_2_ (mL/kg/min)	3.4 ± 1.2	3.6 ± 1.1	.352
V̇CO_2_ (L/min)	0.17 ± 0.05	0.25 ± 0.08	**<**.**001**
V̇E (L/min)	7.1 ± 2.2	8.4 ± 1.8	.065
PETCO_2_ (mmHg)	29 ± 4	30 ± 4	.219
Heart rate (bpm)	70 ± 12	73 ± 10	.394
Gas exchange threshold			
V̇O_2_ (mL/kg/min)	14.5 ± 3.4	11.7 ± 2.7	**<**.**001**
V̇O_2peak_ predicted (%)	63.6 ± 14.2	54.0 ± 13.8	.**011**
V̇CO_2_ (L/min)	0.87 ± 0.26	0.88 ± 0.30	.438
V̇E (L/min)	26.4 ± 6.5	32.4 ± 10.5	.**006**
PETCO_2_ (mmHg)	36 ± 4	34 ± 5	.068
Peak			
V̇O_2_ (mL/kg/min)	24.1 ± 5.7	18.8 ± 4.6	**<**.**001**
V̇O_2_ (L/min)	1.8 ± 0.5	1.5 ± 0.5	.**025**
V̇O_2peak_ predicted (%)	106 ± 19	86 ± 19	**<**.**001**
V̇CO_2_ (L/min)	2.0 ± 0.6	1.6 ± 0.5	.**017**
V̇E (L/min)	67.1 ± 18.7	62.9 ± 19.4	.411
PETCO_2_ (mmHg)	34 ± 4	31 ± 4	.**011**
V̇E/V̇CO_2_ slope	29 ± 4	36 ± 7	**<**.**001**
V̇E/V̇O_2_ ratio	36 ± 6	39 ± 6	.064
Workload (W)	147 ± 52	113 ± 38	.**006**
Heart rate (bpm)	154 ± 16	137 ± 23	**<**.**001**
O_2_ pulse (ml/beat)	12.0 ± 3.0	11.2 ± 3.1	.342
Relative O_2_ pulse (mL/kg/beat)	0.16 ± 0.03	0.14 ± 0.02	.**018**
Recovery			
Heart rate recovery (%)	18 ± 5	12 ± 6	**<**.**001**
Heart rate recovery (bpm)	27 ± 7	18 ± 11	**<**.**001**

V̇O_2_, oxygen uptake V̇CO_2_, volume of expired carbon dioxide; V̇E, volume of expired air per minute; PETCO_2_: CO_2_ partial pressure at the end of expiration also representative of the matching of the ventilation and perfusion. Values in bold indicate significance *p *< 0.05.

Both maximum heart rate and heart rate recovery were lower in kidney transplant recipients than in healthy volunteers. Heart rate recovery (difference between maximal heart rate and heart rate 1 min into recovery) was 12% in kidney transplant recipients compared to 18% in healthy volunteers (*p* < .001). The O_2_ pulse was not significantly different between kidney transplant recipients and healthy volunteers; however, when expressed relative to body weight, O_2_ pulse was significantly lower in kidney transplant recipients (0.16 ± 0.03 vs 0.14 ± 0.02 mL/kg/beat; *p* = .018).

Ventilation rates were similar between groups at rest and at peak exercise, but greater in kidney transplant recipients at gas exchange threshold (26.36 ± 6.48 vs 32.39 ± 10.49 L/min; *p* = **.**006). The V̇E/V̇CO_2_ slope (representative of the matching of ventilation and perfusion) was significantly higher in kidney transplant recipients compared to healthy volunteers (mean difference = 6.63, 95% CI [3.63, 9.63]). The V̇E/V̇O_2_ ratio (reflective of the ventilatory cost of oxygen uptake) was higher in kidney transplant recipients compared to healthy volunteers but this was not significant (39 ± 6 vs 36 ± 6, *p* = .064). The PETCO_2_ response is described in Figure S2 in the Supplemental Materials.

## Discussion

After adjusting for baseline, V̇O₂_peak_ increased by 1.50 mL/kg/min after 12 weeks of home-based rehabilitation. This meets the minimum clinically important difference for non-dialysis chronic kidney disease (CKD)^20^ and reflects a >6% improvement from baseline. In chronic heart failure, each 6% increase in V̇O₂_peak_ correlates with a 5% reduction in risk of all-cause mortality or hospitalisation,^6^ making this change promising in a high cardiovascular risk population. Compared to the two other solely home-based exercise studies in kidney transplant recipients, our findings are notable. Michou et al. reported a 1 mL/kg/min increase after 6 months, with weekly calls and monthly visits.^21^ Painter et al. found no between-group V̇O₂_peak_ differences at 6 months (an increase in both groups occurred likely due post-transplant improvements^22^), but a large 3.6 mL/kg/min at 12 months, likely due to the longer intervention and consistent support.^23^ This is the first trial in transplant recipients to assess detailed cardiopulmonary variables beyond peak values. Although no significant between-group differences were seen at gas exchange threshold or other peak metrics, a ∼1 mL/kg/min improvement in V̇O₂ at threshold moves participants further from the 11 mL/kg/min risk threshold for post-surgical morbidity and mortality.^24^

In Greenwood et al.'s study,^25^ participants did two supervised in-centre sessions and one home-based session weekly for 12 weeks. V̇O₂_peak_ increased by 2.5 mL/kg/min with resistance training and 1.8 mL/kg/min with aerobic training compared to usual care. These gains, achieved through more resource-intensive methods, highlight the value of the present programmes home-based approach. The slight difference in V̇O₂_peak_ may stem from longer aerobic session duration, as intensity was similar. A significant link between total aerobic sessions and V̇O₂_peak_ change suggests volume drives fitness gains. The greater improvement in the resistance group was unexpected but may reflect better tolerance of localised fatigue.

Lack of familiarisation for cardiopulmonary exercise tests could result in differences in data being due to intra-individual variability as opposed to intervention effect.^26^ Secondly, the ECSERT trial was designed as a feasibility and pilot trial and is underpowered to detect differences in cardiopulmonary exercise test measures. As such all-trial data should be considered as requiring confirmation in appropriately powered prospective studies.

This trial compared cardiopulmonary exercise responses between age- and sex-matched kidney transplant recipients and healthy controls in a nested case-control design. Results showed reduced exercise capacity and heightened ventilatory response in transplant recipients, similar to those with mild to moderate CKD,^27^ but better than haemodialysis patients.^28^ Poor cardiopulmonary exercise test outcomes are strongly linked to adverse outcomes and are valuable for risk stratification and rehabilitation programme personaliasation.^17^ Reduced fitness in transplant recipients likely indicates poorer prognosis than in general or chronic disease populations, warranting further studies on clinical outcome associations. These results also justify strongly the need for rehabilitation programmes to be embedded within routine care.

There was reduced oxygen consumption in kidney transplant recipients compared to healthy volunteers at both gas exchange threshold and peak exercise. Oxygen uptake at threshold averaged 11.70 mL/kg/min, just above the 11 mL/kg/min cut-off linked to increased post-surgical risk.^17,24^ Lower V̇O₂ at this point may cause daily activities to rely on anaerobic metabolism, affecting quality of life. Most recipients had V̇O₂peak values in the ‘low’ or ‘fair’ fitness categories, averaging 18.81 mL/kg/min; close to the >17.5 mL/kg/min threshold predictive of survival in end stage kidney disease (ESKD) patients.^29^ Higher V̇O₂peak is linked to greater ability to perform daily activities in chronic heart failure^30^ and hemiparetic stroke,^31^ highlighting its importance beyond high-intensity exercise.

The V̇E/V̇CO_2_ slope represents matching of the ventilation and perfusion within the pulmonary system (V/Q). Values in kidney transplant recipients were above the upper limit of normal,^17,32^ and were significantly higher than in the healthy volunteers and patients living with mild to moderate CKD.^27^ A high V̇E/V̇CO_2_ slope has been linked with negative prognosis in patients living with coronary artery disease,^33^ pulmonary hypertension,^34^ and dilated cardiomyopathy.^35^ In cardiac patients elevated V̇E/V̇CO_2_ slope is associated with increased V/Q mismatching (adequate ventilation and poor perfusion).^32^ The exact mechanisms in kidney transplant recipients and in CKD remain undetermined. Ventilation perfusion mismatching is also suggested by a reduced PETCO_2_ response to exercise; PETCO_2_ should rise to its highest value (usually at gas exchange threshold) and then fall as the individual reaches peak exercise,^32^ which is seen in the present study. A rise of <3 mmHg is considered abnormal; the rise was 4 mmHg in kidney transplant recipients compared to 7 mmHg in healthy volunteers, which is a similar pattern seen in patients with cardiac disease. The ventilatory cost of V̇O_2_ (V̇E/V̇O_2_) was also elevated in kidney transplant recipients. Although this was not significant, the mean value (39) was close to the cut-off value of 40 for normal. This difference was also observed in patients with mild to moderate CKD.^27^ Elevated V̇E/V̇O_2_ is indicative of oxygen delivery far exceeding the utilisation capacity and has been linked to mitochondrial myopathies.^17,36^ Patients living with CKD have skeletal muscle mitochondrial dysfunction,^37^ which has been associated with peripheral limitations to exercise capacity as measured by the six-minute walk test (6MWT).^38^ Not much is known about whether skeletal muscle mitochondrial dysfunction persists post-transplantation, but these results identify a need for further investigation.

A strength of this analysis is the exact age-sex matching between groups. However, limitations include data drawn from two separate studies, making full consistency in testing and analysis difficult despite efforts to align protocols. Spirometry data were not collected to distinguish pulmonary limitations. Participants were recruited for the ECSERT trial, which included an exercise programme. This may have attracted participants already interested in physical activity, potentially biasing results toward a fitter, more motivated sample not representative of all transplant recipients.

Kidney transplant recipients have reduced exercise capacity and increased ventilatory response to exercise compared to healthy volunteers. A home-based combined aerobic and resistance rehabilitation programme may lead to a statistically and clinically meaningful improvement in V̇O_2peak_ back towards normal. Adequately powered studies are warranted to further investigate these hypotheses.

## Clinical Messages

- Kidney transplant recipients demonstrate substantially reduced cardiorespiratory fitness compared to age-sex-matched healthy individuals, indicating persistent cardiovascular vulnerability despite successful transplantation
- Blunted peak exercise responses and impaired heart rate recovery in kidney transplant recipients underscore the need for targeted cardiovascular rehabilitation interventions in this population
- A structured home-based rehabilitation programme could meaningfully improve cardiorespiratory fitness in kidney transplant recipients, supporting its integration into routine post-transplant care
- Higher frequency of aerobic exercise sessions is associated with greater improvements in cardiorespiratory fitness, highlighting the clinical importance of promoting regular aerobic activity post-transplant

## Supplemental Material

sj-docx-1-cre-10.1177_02692155251408792 - Supplemental material for Cardiorespiratory fitness in kidney transplant recipients: A pilot randomised controlled trial of structured home-based rehabilitation and a nested case-control analysis Supplemental material, sj-docx-1-cre-10.1177_02692155251408792 for Cardiorespiratory fitness in kidney transplant recipients: A pilot randomised controlled trial of structured home-based rehabilitation and a nested case-control analysis by Roseanne E Billany, Noemi Vadaszy, Stephanie Burns, Rafhi Chowdhury, Ella C Ford, Zahra Mubaarak, Gurneet K Sohansoha, Jian L Yeo, Abhishek Dattani, Alice C Cowley, Gaurav S Gulsin, Nicolette C Bishop, Alice C Smith, Gerry P McCann and Matthew PM Graham-Brown in Clinical Rehabilitation

sj-doc-2-cre-10.1177_02692155251408792 - Supplemental material for Cardiorespiratory fitness in kidney transplant recipients: A pilot randomised controlled trial of structured home-based rehabilitation and a nested case-control analysis Supplemental material, sj-doc-2-cre-10.1177_02692155251408792 for Cardiorespiratory fitness in kidney transplant recipients: A pilot randomised controlled trial of structured home-based rehabilitation and a nested case-control analysis by Roseanne E Billany, Noemi Vadaszy, Stephanie Burns, Rafhi Chowdhury, Ella C Ford, Zahra Mubaarak, Gurneet K Sohansoha, Jian L Yeo, Abhishek Dattani, Alice C Cowley, Gaurav S Gulsin, Nicolette C Bishop, Alice C Smith, Gerry P McCann and Matthew PM Graham-Brown in Clinical Rehabilitation
