# Supplementary material for: Cardiorespiratory fitness in kidney transplant recipients: A pilot randomised controlled trial of structured home-based rehabilitation and a nested case-control analysis
Source: Clin Rehabil. 2025 Dec 30;40(5):587–602. doi: 10.1177/02692155251408792 (PMC13121817; doi:10.1177/02692155251408792)
Supplement: sj-docx-1-cre-10.1177_02692155251408792 - Supplemental material for Cardiorespiratory fitness in kidney transplant recipients: A pilot randomised controlled trial of structured home-based rehabilitation and a nested case-control analysis [file sj-docx-1-cre-10.1177_02692155251408792.docx]

**SUPPLEMENTARY MATERIAL**

Table S1. Analysis of unadjusted and adjusted mean difference between intervention and control for cardiorespiratory fitness variables at gas exchange threshold

|  | **n** | **Baseline  (mean ± SD)** | **Follow-up** | | **Adjusted mean diff. B-A (CI of Diff)**  **p value (η²)** |
| --- | --- | --- | --- | --- | --- |
|  |  |  | **Unadjusted (mean ± SD)** | **Adjusted (mean ± SE)** |  |
| **Gas exchange threshold** |  |  |  |  |  |
| **V̇O_2_ (mL/kg/min)** |  |  |  |  |  |
| Intervention (B) | 18 | 12.2 ± 3.0 | 13.4 ± 3.2 | 13.5 ± 0.4 | 1.0 (-0.1, 2.1)  0.09 (0.08) |
| Control (A) | 19 | 12.5 ± 2.4 | 12.7 ± 2.7 | 12.6 ± 0.4 |  |
| **V̇O_2_ (L/min)** |  |  |  |  |  |
| Intervention (B) | 18 | 1.01 ± 0.33 | 1.09 ± 0.39 | 1.07 ± 0.03 | 0.07 (-0.02, 0.17)  0.12 (0.07) |
| Control (A) | 19 | 0.99 ± 0.19 | 0.99 ± 0.19 | 1.00 ± 0.03 |  |
| **V̇O_2peak_pred (%)** |  |  |  |  |  |
| Intervention (B) | 18 | 49.8 ± 15.7 | 52.0 ± 15.4 | 53.4 ± 1.6 | 2.4 (-2.1, 6.8) |
| Control (A) | 19 | 51.8 ± 11.9 | 52.2 ± 12.7 | 51.0 ± 1.5 | 0.28 (0.03) |
| **V̇CO_2_ (L/min)** |  |  |  |  |  |
| Intervention (B) | 17 | 0.92 ± 0.34 | 0.99 ± 0.38 | 0.99 ± .05 | 0.08 (-0.05, 0.22)  0.21 (0.49) |
| Control (A) | 18 | 0.95 ± 0.35 | 0.91 ± 0.16 | 0.91 ± 0.05 |  |
| **V̇E (L/min)** |  |  |  |  |  |
| Intervention (B) | 16 | 31.1 ± 11.5 | 33.1 ± 11.9 | 33.1 ± 1.6 | 1.9 (-2.7, 6.4)  0.40 (0.02) |
| Control (A) | 19 | 31.2 ± 9.3 | 31.3 ± 6.0 | 31.3 ± 1.5 |  |

Abbreviations: A, control; B, intervention; GET, gas exchange threshold; pred, predicted; V̇CO_2_, volume of expired carbon dioxide; V̇E, volume of expired air per minute; V̇O_2_, oxygen uptake

Table S2. Cardiorespiratory fitness classification in healthy volunteers and kidney transplant recipients according to the American Heart Association

|  | Cardiorespiratory fitness classification, n (%) | | | | |
| --- | --- | --- | --- | --- | --- |
|  | Low | Fair | Average | Good | High |
| HV | 2 (6.7) | 5 (16.7)* | 15 (50.0) | 8 (26.7)* | 0 (0) |
| KTR | 6 (20.0) | 14 (46.7) | 9 (30.0) | 1 (3.3) | 0 (0) |

Abbreviations: HV, healthy volunteers; KTR, kidney transplant recipients

*p <.05, **p <.0125 between groups


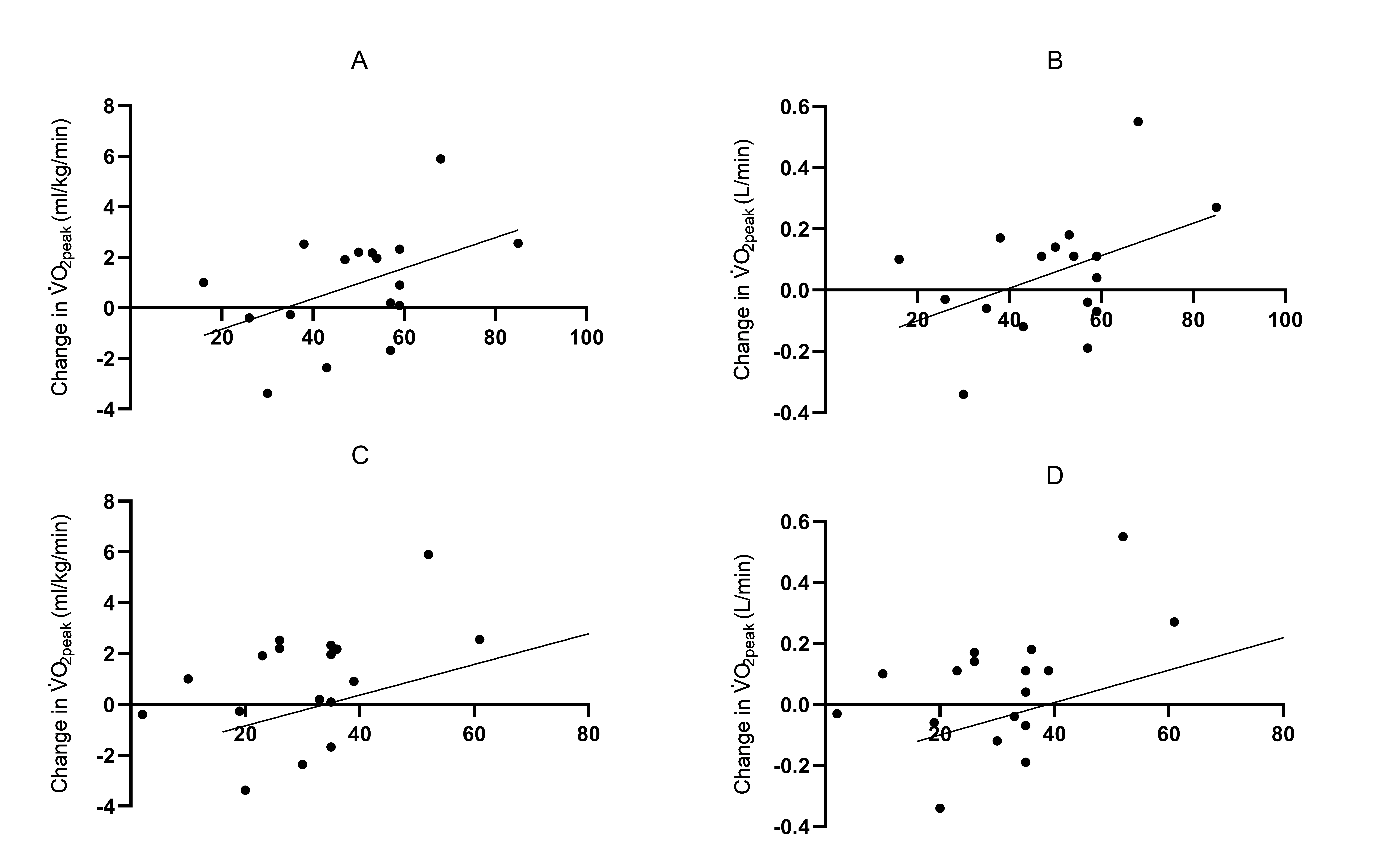


Figure S1: Total number of self-reported exercise sessions across the 12-week intervention and the association with change in cardiorespiratory outcomes following 12 weeks of home-based exercise (total aerobic and resistance sessions; A, V̇O_2peak_ [mL/kg/min]; B, V̇O_2peak_ [L/min]; total aerobic sessions only; C, V̇O_2peak_ [mL/kg/min]; D, V̇O_2peak_ [L/min]).


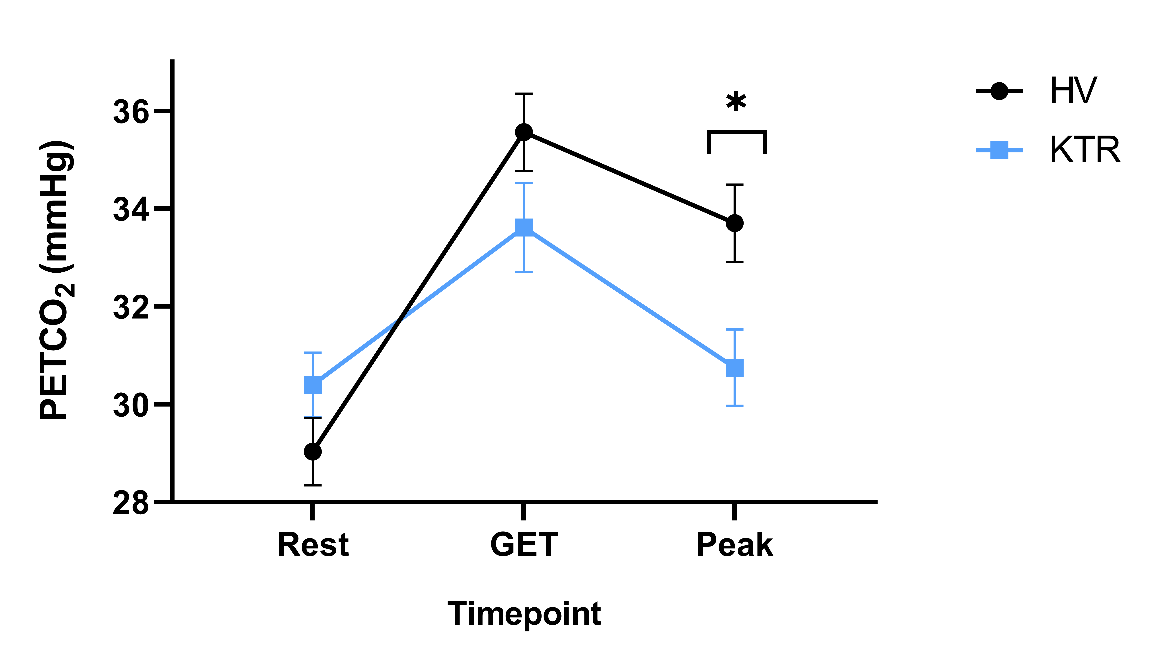
Figure S2. PETCO_2_ at rest, GET, and peak during the CPET.

Abbreviations: GET, gas exchange threshold; CPET, cardiopulmonary exercise test; HV, healthy volunteers; KTR, kidney transplant recipients; PETCO_2_, partial pressure of end-tidal carbon dioxide.

**p* ≤.05 between group

There was a significant group x time interaction for PETCO2 (reflective of the ventilation perfusion relationship) across rest, gas exchange threshold, and peak exercise (p < .001). The response pattern was similar between groups, however PETCO2 did not rise as high in kidney transplant recipients as in healthy volunteers. There was a significant main effect for time (p < .001). Post-hoc analysis showed PETCO2 rose significantly in both groups between rest and gas exchange threshold (p < .001). There was a significant main effect for group (p < .001) and post-hoc analysis showed there was a significant between group difference at peak exercise only. Both groups showed a drop in PETCO2 between gas exchange threshold and peak exercise, however this was only significant in kidney transplant recipients (healthy volunteers -1.89 ± .79, p = .071 vs kidney transplant recipients -2.86 ± .42 mmHg, p < .001).
